# Rostral Intralaminar Thalamus Engagement in Cognition and Behavior

**DOI:** 10.3389/fnbeh.2021.652764

**Published:** 2021-04-15

**Authors:** Kara K. Cover, Brian N. Mathur

**Affiliations:** Department of Pharmacology, University of Maryland School of Medicine, Baltimore, MD, United States

**Keywords:** thalamostriatal, basal ganglia, consciousness, cognitive control, attention, thalamocortical, memory

## Abstract

The thalamic rostral intralaminar nuclei (rILN) are a contiguous band of neurons that include the central medial, paracentral, and central lateral nuclei. The rILN differ from both thalamic relay nuclei, such as the lateral geniculate nucleus, and caudal intralaminar nuclei, such as the parafascicular nucleus, in afferent and efferent connectivity as well as physiological and synaptic properties. rILN activity is associated with a range of neural functions and behaviors, including arousal, pain, executive function, and action control. Here, we review this evidence supporting a role for the rILN in integrating arousal, executive and motor feedback information. In light of rILN projections out to the striatum, amygdala, and sensory as well as executive cortices, we propose that such a function enables the rILN to modulate cognitive and motor resources to meet task-dependent behavioral engagement demands.

## Introduction

The mammalian thalamus can be parcellated into ~60 nuclei defined by cytoarchitecture and connectivity properties (Jones, 2007). Examination of afferent and efferent connections reveals several organizational themes among the nuclei. The first, and perhaps most studied, grouping is the first-order sensory relay nuclei. These regions receive inputs from peripheral sensory systems and faithfully transmit information to the corresponding primary sensory cortical region through direct glutamatergic synapses. The lateral geniculate nucleus of the thalamus, for example, relays visual information from the retina to the primary visual cortex.

A second class of thalamic nuclei are referred to as higher-order association nuclei. These relay nuclei are noted for being innervated by a primary sensory cortical area and, in turn, project to the corresponding secondary sensory cortical region. For example, a primary target of the visual cortex is the thalamic pulvinar nucleus which, in turn, serially innervates higher order visual cortical areas to facilitate spatial attention through synchronization of visual cortical areas (Saalmann et al., 2012). Other proposals for the function of such cortico-thalamo-cortical (or trans-thalamic) pathways suggest roles in entraining otherwise isolated cortical regions, providing efference copies to subcortical systems, or serving as a coincidence detector for parallel cortico-cortical signaling (Sherman, 2016). Another example of higher-order association nuclei in the thalamus is the reticular nucleus. Enveloping the lateral boundary of the thalamus, this GABAergic cellular group receives axon collaterals from passing thalamo-cortical and cortico-thalamic projections and innervates nearly all thalamic nuclei. These circuits enable feedforward and feedback inhibitory circuits to modulate thalamocortical signaling, as well as exert lateral inhibition across otherwise disconnected thalamic nuclei with limited inhibitory microcircuitry (Crabtree, 2018).

**Figure 1:**
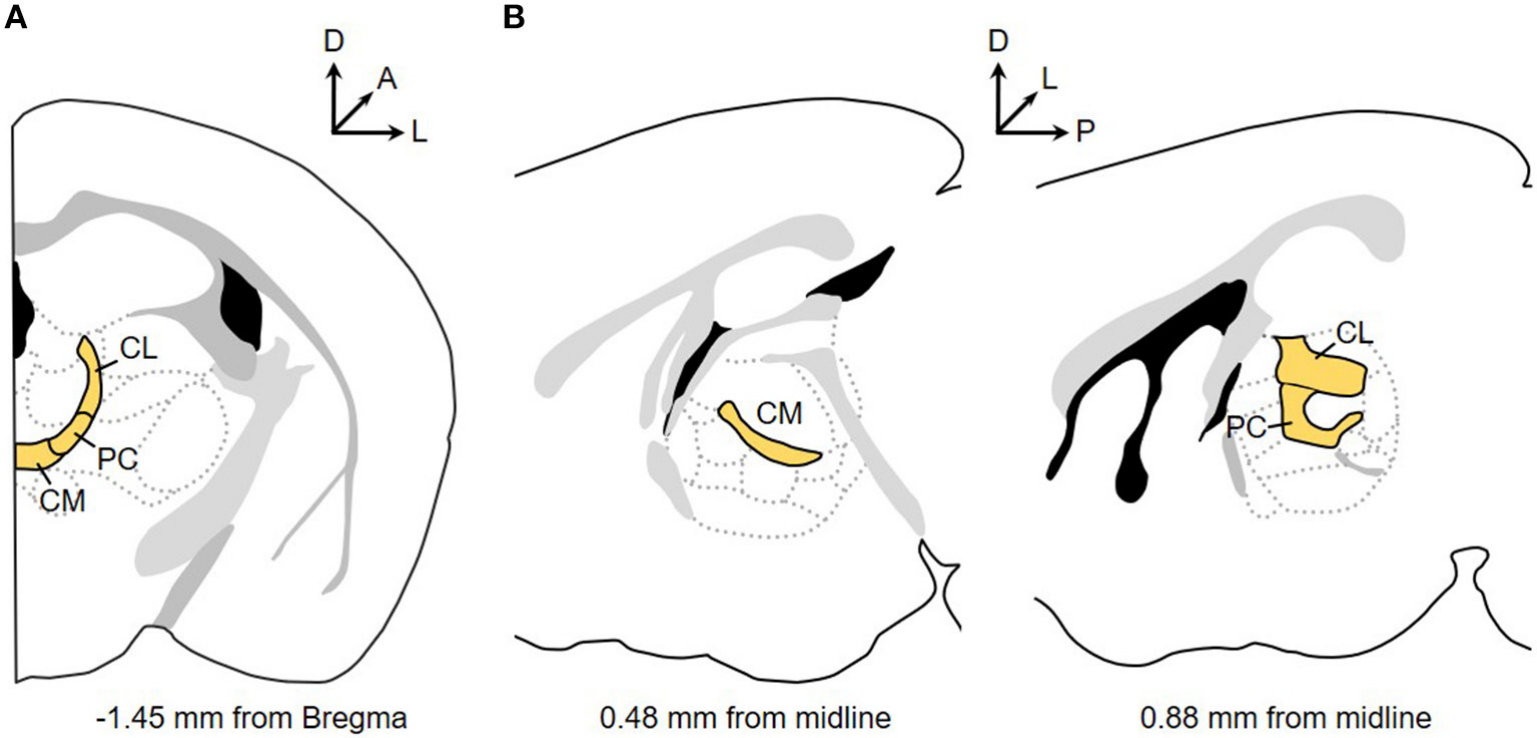
The mouse rostral intralaminar nuclei (rILN). Coronal **(A)** and sagittal **(B)** perspectives of the central lateral (CL), paracentral (PC), and central medial (CM) nuclei. Arrows indicate orientation of dorsal (D), lateral (L), anterior (A), and posterior (P) planes.

The final group of thalamic nuclei are located on the midline or nestled within the internal medullary lamina. This grouping is referred to as the “non-specific” thalamus for the long-held, but since, challenged view that these nuclei globally activate the cortex (Groenewegen and Berendse, 1994). Along the midline are the paraventricular, intermediodorsal, paratenial, reuniens, rhomboid, and in primates, subfascicular nuclei (Jones, 2007). More caudally, the medullary lamina splits and contains the parafascicular nucleus (Pf), and more laterally, the centré median nucleus (referred to here as CeM). The boundary distinguishing these two nuclei is undetectable in rodents and other smaller mammals; thus, the posterior intralaminar nuclei are referred to solely as the Pf in these species with the consideration that the lateral component of this nucleus is homologous to the CeM (Jones, 2007). Located anteriorly within the lamina are the rostral intralaminar nuclei (rILN): the central lateral (CL), paracentral (PC), and central medial (CM) nuclei. In the rodent, these three nuclei are parceled from a continuous band of neurons spanning from the midline, curving around the ventrolateral boundary of the mediodorsal nucleus and terminating ventral to the hippocampal dentate gyrus and lateral to the lateral habenula (Figure 1) (Franklin and Paxinos, 2008). Delineating the boundaries of the PC is difficult, which is usually defined by the more flattened appearance of cells compared to the adjacent medially-located CM and dorsally-positioned CL. This general structure of the rILN is preserved in the cat but disrupted and discontinuous in the primate (Jones, 2007).

In the following sections we review rILN anatomical connectivity, examine how these connections confer roles for these nuclei within specific functional domains, and assess potential involvement of the rILN in multi-system disease states. Finally, we present a conceptual framework describing how these thalamic nuclei contribute to a wide array of behavioral functions. Our review primarily draws from studies conducted in rodents. However, we note findings derived from other species where appropriate.

## rILN Anatomical Connectivity

**Figure 2:**
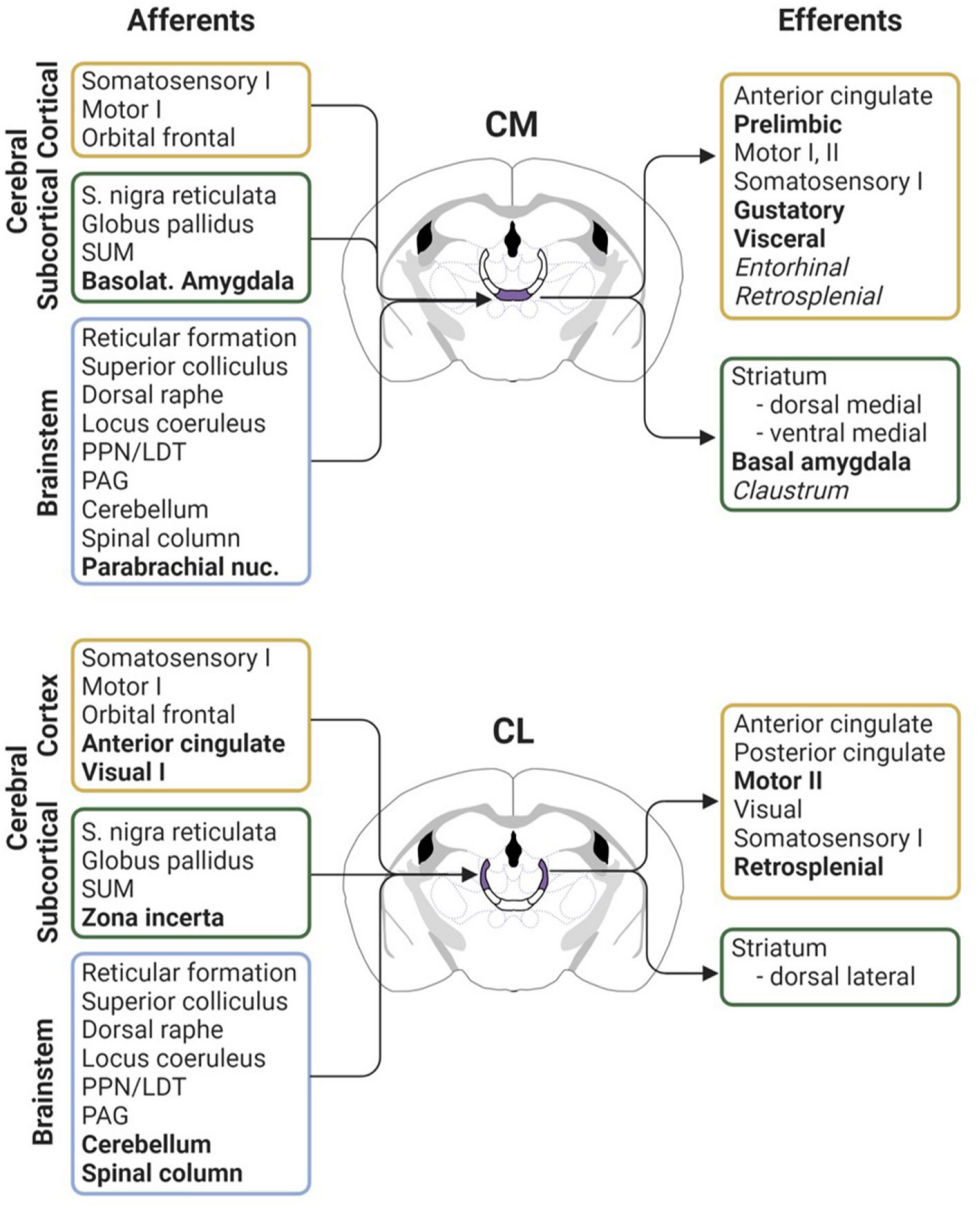
Comparative anatomical connectivity of the rodent CM and CL intralaminar nuclei. Afferent inputs (left) and efferent outputs (right) for the CM (top; purple) and CL (bottom; purple) nuclei. Bolded text indicates notably denser or exclusive projections as compared between the two thalamic nuclei. Italicized text denotes sparse projection innervation. Figure created with BioRender.com. Connectivity derived from: Krettek and Price (1977), Wise and Jones (1977), Carter and Fibiger (1978), Beckstead (1979), Wang et al. (1999), Ichinohe et al. (2000), Krout and Loewy (2000), Barthó et al. (2002), Krout et al. (2002), Van der Werf et al. (2002), Vertes et al. (2012), Rizzi and Tan (2019), Huerta-Ocampo et al. (2020), and Prasad et al. (2020). LDT, laterodorsal tegmental nucleus; PAG, periaqueductal gray; PPN, pedunculopontine nucleus; SUM, supramammillary nucleus; I, primary cortex; II, secondary cortex.

Like the relay thalamic nuclei, the intralaminar nuclei are primarily composed of glutamatergic projection neurons. A notable difference from thalamic relay nuclei, however, is the breadth of afferents that arise from sensory, motor, and limbic modalities to innervate the rILN (Figure 2). The rILN (and Pf) are predominately innervated by subcortical areas. Major excitatory afferents to the rILN include the superior colliculus, hypothalamic supramammillary nucleus, reticular formation, parabrachial nucleus, and deep cerebellar nuclei, as well as several first- and higher- order thalamic nuclei (Krout and Loewy, 2000; Krout et al., 2001, 2002). Whereas both the rILN and the Pf receive input from the cortex, the rILN are notably innervated by a wider range of cortical regions including cingulate, retrosplenial, parietal, insula, prefrontal, somatosensory, supplementary motor, auditory, and visual cortices (Van der Werf et al., 2002; Prasad et al., 2020). In contrast, only the frontal and parietal cortices innervate the Pf (Cornwall and Phillipson, 1988).

Inhibitory inputs to the rILN arise from the substantia nigra pars reticulata, habenula, zona incerta, thalamic reticular nucleus, and the external segment of the globus pallidus (Carter and Fibiger, 1978; Van der Werf et al., 2002; Rizzi and Tan, 2019). The rILN are also innervated by a range of modulatory inputs including the periaqueductal gray, cholinergic pontine and tegmental nuclei, noradrenergic locus coeruleus, and serotonergic raphe nuclei (Van der Werf et al., 2002; Huerta-Ocampo et al., 2020). Accordingly, the rILN are enriched in metabotropic receptors as compared to the thalamic relay neurons (Phillips et al., 2019). Together, this compilation of anatomically, neurochemically, and functionally diverse efferents endows the rILN as distinct thalamic integrators of inputs from many cortical and subcortical cerebral centers.

Examination of rILN efferents reveals a pattern of projections distinct from that of both thalamocortical relay neurons and the Pf. Whereas, the thalamic relay nuclei generally target cortical regions related to a specific sensory or functional modality and the Pf weakly projects to a restricted number of cortical areas, the rILN defy cortical functional boundaries and innervate widely. Neural circuit-specific investigation reveals subtle differences in innervation patterns between the rILN nuclei that are most apparent when comparing the CL and CM (Figure 2). Collectively, these nuclei send excitatory projections to cingulate, agranular insula, lateral orbital, parietal, retrosplenial, entorhinal, frontal eye field, gustatory, visceral, auditory, visual, motor, and somatosensory cortices (Yanagihara et al., 1987; Berendse and Groenewegen, 1991; Van der Werf et al., 2002).

The intralaminar nuclei also differ from the thalamic relay nuclei in their innervation of subcortical regions. In particular, these nuclei densely innervate the striatum. All three nuclei of the rILN project to the entirety of the striatal complex (Van der Werf et al., 2002). These projections are loosely topographically organized with the laterally-positioned CL most densely innervating the dorsolateral striatum and the medially-located CM targeting the dorsomedial striatum. Although the nucleus accumbens receives denser input from adjacent midline paraventricular and intermediodorsal nuclei, both the CM and PC innervate this ventral region as well (Van der Werf et al., 2002). The caudally-lying Pf, in comparison, innervates the striatum more densely (Mandelbaum et al., 2019). Whereas projections generally span the majority of the striatum, the Pf more strongly innervates the dorsolateral striatum and nucleus accumbens (Sadikot et al., 1992; Van der Werf et al., 2002).

How does rILN anatomical connectivity compare to that of the other non-specific thalamic nuclei? The rhomboid and reuniens nuclei are notably reciprocally connected with the medial prefrontal cortex and hippocampus, suggesting roles in higher-order cognitive processes (Cassel et al., 2013). The paraventricular, paratenial, and intermediodorsal nuclei are innervated by a range of brainstem structures and primarily project to the ventral striatum, medial prefrontal cortex, and amygdala; connectivity that is proposed to contribute to viscerosensory awareness and motivation (Van der Werf et al., 2002; Millan et al., 2017). In comparison, the rILN appear to be innervated by a wider range of cortical and brainstem regions in addition to uniquely projecting to the cingulate cortex and dorsal striatum.

rILN afferent and efferent connectivity reveal additional anatomical patterns that may inform function. First, examination of thalamocortical and thalamostriatal projections shows that the rILN innervate striatal areas that are also targeted by cortical regions that the rILN also directly innervate and/or receive cortico-thalamic projections (Hunnicutt et al., 2016). For example, CM projections to the striatum converge with prelimbic and medial orbital corticostriatal projections; CM projections also directly terminate in prelimbic and medial orbital cortices (Hunnicutt et al., 2016). These connectivity patterns may serve to synchronize or entrain thalamic and cortical components of functional systems (e.g., motor, limbic, or sensory) to guide basal ganglia output activity. Second, the rILN are innervated by the basal ganglia output nucleus, the substantia nigra pars reticulata, and are also innervated by nuclei that receive inputs from the substantia nigra pars reticulata, including the superior colliculus, pedunculopontine nucleus, and reticular formation. Through rILN innervation of the striatum, these thalamic nuclei may complete subcortical- basal ganglia re-entrant loops. Whereas cortico-striatopallidal-thalamocortical loops (Alexander et al., 1986; Aoki et al., 2019; Lee et al., 2020) are proposed to facilitate action-outcome learning (Redgrave et al., 2011), consideration for subcortical- basal ganglia loops has been largely overlooked until relatively recently (McHaffie et al., 2005). Functional validation of such loops may uniquely implicate the rILN in a range of basal ganglia -mediated behaviors.

rILN projections exhibit notable differences in cortical synaptic targets, as compared to the thalamic relay nuclei. First-order thalamic relay axons terminate in middle cortical layers whereas higher-order nuclei innervate superficial layers (Jones, 2001). In contrast, rILN projections terminate in superficial (I), middle (III), and deep (V) cortical layers (Van der Werf et al., 2002; Unzai et al., 2017). Whereas, the functional significance of this innervation pattern is unknown, this arrangement may enable robust and coordinated activation of targeted cortical columns. In agreement, high frequency rILN stimulation induces c-fos expression spanning cortical layers II though VI (Shirvalkar et al., 2006).

In the striatum, comparison of rILN and Pf afferent synaptic morphology reveals striking differences between the two thalamic projections. rILN terminals form axo-spinous synapses on striatal medium spiny neurons (MSNs) (Raju et al., 2006) that induce large facilitative AMPA receptor -mediated responses (Ellender et al., 2013). Pf axons, in contrast, synapse on MSN dendritic shafts and induce relatively weaker NMDA receptor -mediated responses characteristic of a more modulatory influence on MSN signaling (Lacey et al., 2007; Ellender et al., 2013). Moreover, rILN axons sparsely arborize in the striatum. Their long axon collaterals contact many MSNs through *en passant* boutons, whereas Pf neurons form dense clusters of terminals to focally converge on fewer neurons (Deschênes et al., 1995). Together, these properties may enable the rILN to effectively drive MSN output signaling across larger volumes of the striatum. Of note, these studies exclusively examined neurons within the CL nucleus. It remains to be determined whether the PC and CM share similar synaptic properties in their striatal terminations.

## Physiological Features of the rILN

The extensive connectivity of the rILN with brainstem, basal ganglia, and cortical regions distinguishes these nuclei from the primarily unimodal thalamic relay nuclei. Further distinguishing the rILN are unique physiological features. In awake monkeys, cats, and mice, rILN neurons exhibit tonic single-spike firing at 6–8 Hz (Glenn and Steriade, 1982; Gent et al., 2018; Redinbaugh et al., 2020). Sensory events evoke transient burst firing and the majority of rILN neurons exhibit changes in firing rate correlating to eye position or saccadic activity (Schlag and Schlag-Rey, 1984; Schlag-Rey and Schlag, 1984; Wyder et al., 2003). In relation to oculomotor saccades, some cells exhibit pause-rebound firing with pauses in firing occurring during or following saccadic activity; other rILN neurons burst fire prior to or during saccadic activity and are typically selective for a saccade direction in cats (Schlag-Rey and Schlag, 1984). In primates, rILN neurons frequently increase firing during delay periods in sensory-cued reaction time tasks (Wyder et al., 2003; Schiff et al., 2013).

rILN firing activity is governed by sleep-wake states. During non-REM sleep or under anesthesia, tonic firing diminishes as rILN neurons predominately fire in short infrequent bursts (3–6 spikes at 300–600 Hz with inter-burst intervals of 3–10 Hz) that correspond with cortical slow wave activity at relatively hyperpolarized membrane potentials (Glenn and Steriade, 1982; Lacey et al., 2007; Redinbaugh et al., 2020). Depolarizing low-threshold calcium spikes facilitate the brief action potential bursts, which are notably less prevalent in Pf neurons (Brunton and Charpak, 1998; Lacey et al., 2007). rILN burst firing is phase-advanced to the onset of slow wave sleep up-states. Mimicking these firing bursts through optogenetic rILN activation enhances slow wave activity suggesting a causal role for the rILN in driving cortical synchrony during sleep (Gent et al., 2018). rILN activity during REM sleep is generally similar to that of wake states in regard to both tonic and burst firing rates (Glenn and Steriade, 1982).

A distinct population of neurons was identified in the cat dorsal CL characterized by larger cell bodies and significantly faster sleep-associated firing frequencies of 800–1,000 Hz spike bursts. Moreover, this bursting activity is largely preserved in REM sleep and awake states (Steriade et al., 1993). These faster firing rates notably correlate with optimal CL stimulation frequencies to induce wakefulness in primates (Redinbaugh et al., 2020) and rats (Liu et al., 2015), suggesting that the rILN causally facilitate awareness through activation of cortical or subcortical targets.

## Functional Attributes of the rILN

Given their extensive anatomical connectivity, it is not surprising that the rILN are associated with a wide range of behavioral functions. Broadly, these nuclei are implicated in consciousness, sensory and pain processing, executive function, and action control. We review the anatomical and behavioral evidence for each of these functions below.

### Consciousness and Arousal

Heavily innervated by the reticular formation, the rILN were historically considered to serve as a continuation of the ascending reticular activating system (ARAS): the series of brainstem-located nuclei responsible for regulating sleep-wake states. rILN neuronal activity shifts from tonic to burst firing in the transition from sleep to wake states (Glenn and Steriade, 1982). Correspondingly, electrical stimulation of the feline rILN induces a so-called “recruiting response” of slow wave activity that spans much of the cortex (Morison and Dempsey, 1941). Although similar responses may also be evoked through stimulation of various higher-order thalamic neurons, the rILN endure as a target in clinical applications. While rILN damage is associated with cognitive impairment and disorders of consciousness (Schiff, 2008), deep brain stimulation targeting this region demonstrates therapeutic efficacy in patients in chronic minimally conscious states (Schiff et al., 2007; Giacino et al., 2012). In examination of the mechanisms mediating consciousness, rILN activation that accompanies sleep to wake transitions increases cortical deep layer firing rates in the lateral intraparietal area and modulates synchrony between this region and the frontal eye field in primates (Redinbaugh et al., 2020). rILN activation similarly induces rapid wakefulness in sleeping mice and enhances global cortical synchrony through local activation of the cingulate cortex that propagates to posterior cortices through a dorsal thalamic relay (Gent et al., 2018). Thus, these nuclei may promote arousal through coordinated cortical activation.

Abnormal regulation of arousal by the rILN may underlie other pathological conditions. Individuals with temporal lobe epilepsy, the most common form of epilepsy, exhibit increased connectivity between the rILN and ARAS brain structures and the occipital lobe (González et al., 2019). Chemogenetic suppression of the rILN blocks seizure activity in a rodent model of epilepsy (Wicker and Forcelli, 2016) and deep brain stimulation targeting adjacent thalamic structures reduces seizure frequency in patients (Li and Cook, 2018). Together, these findings suggest that the rILN participate in seizure propagation through abnormal connectivity with cortical and arousal-regulating brain regions.

### Cognition

#### Learning

The high connectivity of the rILN with brain structures comprising the limbic system enables these nuclei to influence cognitive processes (Yanagihara et al., 1987; Vertes et al., 2015). Assessments of rILN contributions to learning reveal conflicting results. For example, rodents with rILN lesions show intact learning ability in finding a hidden platform over multiple trials in the Morris water maze in one study (Lopez et al., 2009), but exhibited significant impairments in another (Mair et al., 1998) despite similar experimental parameters. Manipulations specifically inhibiting the rILN to striatum pathway demonstrate intact ability to learn a two-lever appetitive operant task, but pronounced impairments in reversal learning (Kato et al., 2018). Future interrogation of specific rILN projection circuits are likely to determine unique contributions to specific types of sensory and motor learning.

#### Memory

Matching-to-sample or position tasks assess sensory discrimination with versions that implement a delay prior to the response period to test working memory. Lesioning the rILN produces impairments on delayed spatial or olfactory discrimination tests indicating a deficit in working memory, but not sensory discrimination (Mair et al., 1998; Zhang et al., 1998). Moreover, electrical rILN stimulation improves performance when delivered during the delay or response period of the delayed matching-to-position task, further implicating the rILN in working memory and retrieval processes (Mair and Hembrook, 2008). rILN-lesioned rats successfully complete radial arm mazes in the presence of spatial cues but show significant deficits when forced to use an egocentric navigation strategy (Mair et al., 1998; Mitchell and Dalrymple-Alford, 2006). Together, these experiments demonstrate a consistent role for the rILN in working memory. This functional process is likely mediated through rILN projections to the cortex, as selective elimination of the rILN thalamostriatal pathway does not impair spatial working memory (Kato et al., 2018).

The rILN contribute to other memory processes. High frequency rILN stimulation enhances object recognition memory following a 2-hour delay between first object interaction and re-testing for recognition of that object and induces transcription of zif268, an immediate early gene upregulated during long-term potentiation, in the anterior cingulate cortex and hippocampal dentate gyrus (Shirvalkar et al., 2006). In an assessment of spatial long-term memory function, rILN-lesioned rats successfully recall the location of a hidden platform in the visual-cued Morris water maze 5 days, but not 25 days, following acquisition (Lopez et al., 2009). Whether this deficit in remote spatial memory is due to impaired memory formation or retrieval remains unclear.

The rILN are also susceptible to pathology in cognitive disorders marked by memory impairment. Alpha-synuclein deposits form in the rILN in individuals with Parkinson's disease or Lewy Body Dementia (Brooks and Halliday, 2009). The functional consequence of this pathology is unclear, but may contribute to cognitive impairment observed in Parkinsonian patients. In a beta-amyloid model of Alzheimer's disease, rILN stimulation rescues both spatial memory deficits and dendritic regression in the prefrontal cortex and hippocampus (Tsai et al., 2020). Given that the rILN innervate brain regions mediating saccade initiation (frontal eye fields) as well as working memory and attention (prefrontal cortex and posterior parietal cortex), these thalamic nuclei may govern multiple functions supporting cognition.

### Sensory-Related Attention

The involvement of the rILN in arousal naturally extends to attentional processes. Abnormal rILN connectivity with ARAS-regulating brainstem structures correlates with deficits in visuospatial attention in humans (González et al., 2019). Visual or somatosensory -cued transitions from relaxed to attention-demanding states correspond to increased (non-specific) intralaminar nuclei activity (Kinomura et al., 1996). Moreover, rILN firing during a sensory-cued reaction time task correlates to performance. Incorrect responses during this task correspond to smaller increases or less sustained shifts in rILN firing during the cue-response delay, suggesting a role for these nuclei in attentional effort (Schiff et al., 2013). Unilateral rILN lesions in cats commonly result in contralateral visual neglect (Orem et al., 1973) and bilateral lesions in rats produce deficits in spatial orientation (Jeljeli et al., 2000). Correspondingly, non-specific intralaminar nuclei stimulation in cats induces eye movement and improves perception during visual orientation (Hunsperger and Roman, 1976).

The rILN receive glutamatergic input from the intermediate and deep layers of the superior colliculus (Krout et al., 2001). This pathway provides a source for multimodal sensory information to the rILN (Stein and Meredith, 1993). Accordingly, rILN responses are not limited to visual stimuli. These nuclei also fire in response to auditory tones and touch (Grunwerg and Krauthamer, 1992; Sanford et al., 1992). Given the presence of dedicated thalamocortical relay circuits for processing specific sensory modalities, it is unlikely that the rILN directly contribute to sensory perception. Rather, these thalamic nuclei are hypothesized to facilitate attentional engagement of sensory events (Groenewegen and Berendse, 1994; Schiff et al., 2013). One function for such a role is to prepare for motor responding. Indeed, rILN firing associated with self-initiated and visually-cued eye movements commences prior to the movement (Schlag-Rey and Schlag, 1984). rILN neurons rarely encode sensory cues without also exhibiting saccade-related activity (Wyder et al., 2003), suggesting that this region may participate in the transformation of sensory signals into motor commands (Wyder et al., 2004). Alternatively, saccade-related rILN activity may serve to facilitate visuospatial awareness by priming cortical areas for processing new information that follows execution of the saccade (Purpura and Schiff, 1997).

### Pain

In addition to sensory-evoked activity, rILN neurons fire in response to a range of noxious stimuli with large receptive fields (Zhang and Zhao, 2010; Deng et al., 2020). Connectivity with both brainstem and limbic structures positions this thalamic region to participate in pain processing. Several afferents are proposed to relay pain information to the rILN including the glutamatergic ventrolateral periaqueductal gray (Deng et al., 2020) and ipsilateral spinoparabrachial pathway (Deng et al., 2020). Additionally, the rILN are directly innervated by the spinal cord (Wang et al., 1999) and the trigeminal nerve (Sato et al., 2020). Chemically inhibiting the rILN alleviates behavioral responses to mechanical allodynia (Sun et al., 2020) and local activation of 5-HT1A/7 receptors reduces response to tail shock (Harte et al., 2005).

The rILN are hypothesized to mediate the emotional and motivational aspects of pain (Sewards and Sewards, 2002) and may potentially do so through reciprocal connectivity with the basolateral and central amygdala (Krettek and Price, 1977; Vertes et al., 2012; Deng et al., 2020; Sun et al., 2020). Moreover, μ-opioid receptors are highly expressed in the rILN (Mansour et al., 1994). Receptor activation hyperpolarizes rILN neurons and shifts activity from tonic to constrained burst firing (Brunton and Charpak, 1998), providing a pharmacological target for modulating the pain response. Lastly, morphine administration induces greater c-fos expression in the male rat rILN as compared to females (D'Souza et al., 1999). Although the behavioral significance of this finding requires further study, it may implicate the rILN in mediating sex differences observed in endogenous and μ-opioid activated analgesia in humans (Wiesenfeld-Hallin, 2005).

## Clinical Correlates of rILN Function

The extensive anatomical connectivity and involvement of the rILN in a range of behaviors suggest that these nuclei may participate in disorders spanning multiple functional systems. Schizophrenia presents a constellation of symptoms encompassing sensory, motor, and cognitive dysfunction (Delevoye-Turrell et al., 2007; Hartmann et al., 2015; Morris et al., 2018; Wilquin et al., 2018; Culbreth et al., 2020). Whereas, hyperactive striatal dopamine signaling may contribute to pathology (Abi-Dargham et al., 2009; Sekiguchi et al., 2019), evidence also exists for non-specific thalamic hypofunction. Schizophrenic patients exhibit thalamic structural alterations with reduced volume and glutamate receptor expression, in addition to altered thalamic connectivity with cortex and striatum (Watis et al., 2008; Steullet, 2019). Thalamic hypoactivity is observed in patients who perform poorly on oculomotor tasks requiring cognitive control (Camchong et al., 2006). These cognitive deficits are presumed to be mediated by reduced mediodorsal thalamus to PFC signaling based on neuroimaging methods (Huang et al., 2019). Given that the rILN lie immediately adjacent to this nucleus and project to the PFC, the rILN may also participate in schizophrenia pathology. In support of rILN hypofunction in schizophrenia, elimination of NMDA receptors from the intralaminar nuclei induces deficits in working memory, spatial memory, and attention in mice (Yasuda et al., 2017), which are cognitive deficits characteristic of schizophrenia (Mohamed et al., 1999). These animals also exhibit altered sleep patterns and reduced cortical oscillatory activity; symptoms that are also present in schizophrenia (Chan et al., 2017). The induction of this phenotype by loss of rILN NMDA receptors suggests that loss of excitatory drive onto the rILN may contribute to some of the negative symptoms and cognitive deficits observed in schizophrenia.

The rILN are innervated by motor centers including the reticular formation and cortical supplementary motor area and, in turn, project to both the primary motor cortex and striatum. High frequency rILN stimulation produces general increases in locomotion (Shirvalkar et al., 2006). Conversely, lesions result in delayed initiation of goal-directed actions (Burk and Mair, 2001) and impaired motor coordination (Jeljeli et al., 2000). Chemogenetic suppression of rILN signaling causes decreases in spontaneous locomotion (Cover et al., 2019) and optogenetic inhibition produces motor cessation (Giber et al., 2015). rILN innervation of the striatum is specifically implicated in a range of action-related functions. The rILN relay excitatory signaling arising from the cerebellar dentate nucleus to the striatum (Chen et al., 2014) and contribute to motor coordination (Sakayori et al., 2019). Eliminating thalamostriatal glutamate release impairs motor coordination, further emphasizing the influence of this projection on action expression (Melief et al., 2018). Virally lesioning striatal-projecting rILN neurons degrades behavioral flexibility and switching between learned actions (Kato et al., 2018). Together, these findings emphasize a multifaceted role for the rILN in action execution. Therefore, these nuclei may contribute to disease states characterized by generalized disordered actions, such as Attention Deficit Hyperactivity Disorder (Jones et al., 2020).

Recent studies report that the rILN evoke dopamine release in the striatum through a di-synaptic circuit involving striatal cholinergic interneurons. Specifically, activation of rILN terminals synapsing on striatal cholinergic interneurons results in local striatal dopamine release (Cover et al., 2019). This is enabled by downstream striatal cholinergic interneuron innervation of nigrostriatal dopamine terminals (Cachope et al., 2012; Threlfell et al., 2012). Optogenetic activation of striatal rILN terminals is behaviorally reinforcing in a dopamine D1 receptor -dependent manner, demonstrating that this local dopamine release mechanism is functionally significant (Cover et al., 2019). Taken together, these findings stand to implicate the rILN in a range of action and cognitive-related behaviors associated with striatal dopamine signaling. Substance abuse, for example, is marked by pathological execution of maladaptive and harmful actions. In an animal model of methamphetamine self-administration, rILN-mediated incubation of drug craving is dependent on striatal D1-receptor signaling (Li et al., 2018). Moreover, increased midline and intralaminar thalamic activity is associated with cue-evoked craving and physiological arousal in alcohol drinkers (Wang et al., 2019). Understanding how drugs of abuse influence rILN signaling may thus reveal novel therapeutic targets for addiction treatment.

## Discussion

**Figure 3:**
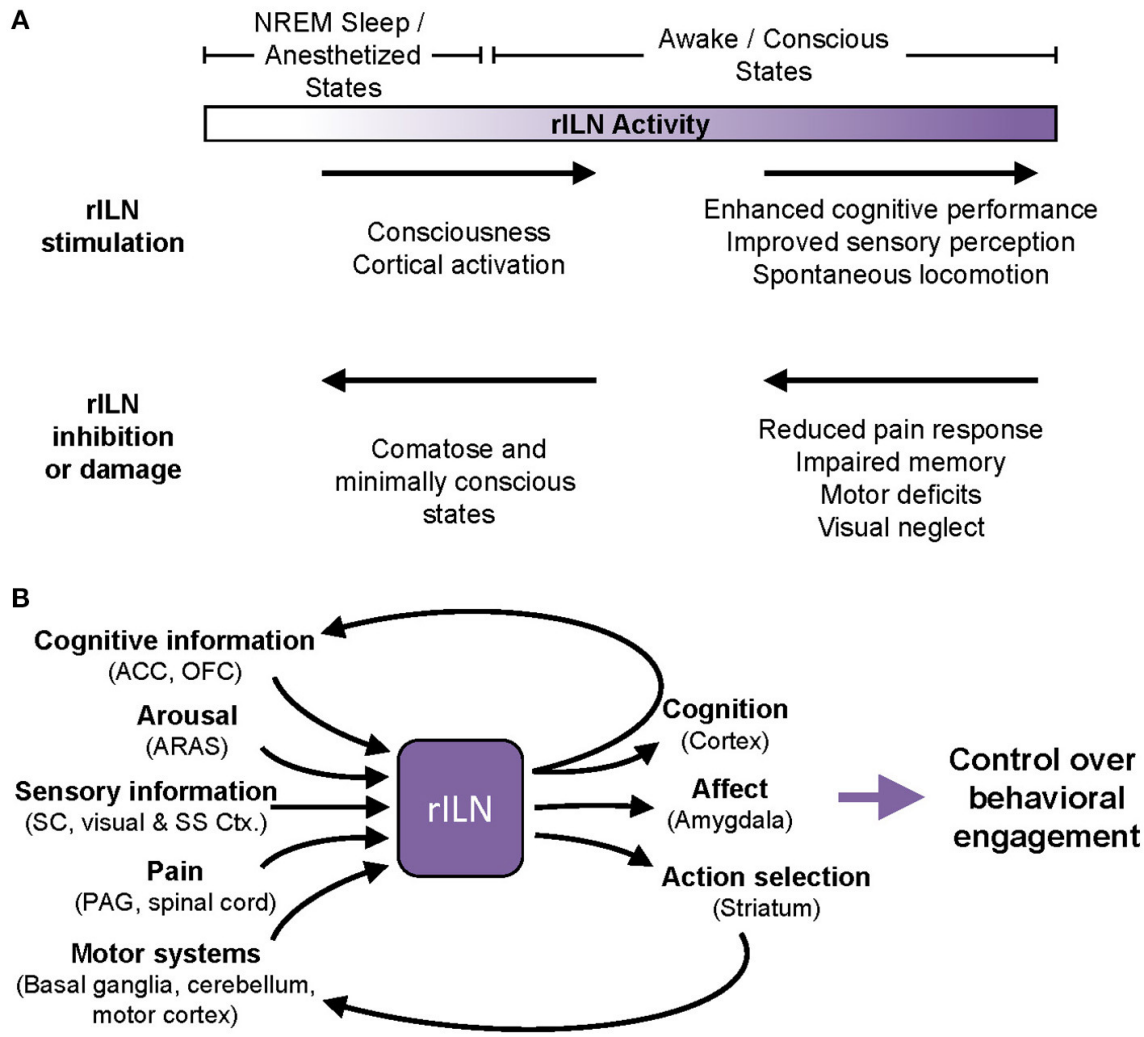
A proposal for modulation of cognitive and behavioral engagement by the rILN. **(A)** Clinical and experimental evidence demonstrate that rILN activity modulates behavioral processes. Minimal rILN activity (left) occurs during sleep or under anesthesia, whereas heightened rILN function (right) is associated with consciousness and optimal attentional states. Within this spectrum of activity, modulations to rILN function induce bi-directional changes in sensory perception, executive function, and motor control. **(B)** The rILN are anatomically positioned to regulate behavioral engagement. The rILN receive information related to cognitive control and decision-making, arousal, sensory information, pain, and motor function (left). Integrating these diverse signals, the rILN may drive task-relevant gains in cognitive and action control through excitation of efferent processes including cognitive networks, affective responses, and action execution (right). ACC, anterior cingulate cortex; ARAS, ascending reticular arousal system; Ctx, cortex; NREM, non-REM; OFC, orbitofrontal cortex; SC, superior colliculus; SS, somatosensory.

*In vivo* recordings demonstrate that the rILN are driven by ARAS activity. Accordingly, rILN firing activity and rILN-induced cortical activation are strongly modulated by sleep and wake arousal states. However, the rILN are not a simple continuation of ARAS; rILN reciprocal connectivity with cortical regions and the basal ganglia elevates this thalamic center to a higher-order integration center. This is supported by the behavioral evidence that rILN activation globally enhances consciousness, memory function, and perceptual decision-making. Conversely, negatively modulating rILN activity broadly impairs motor function, sensory perception, and cognitive ability (Figure 3B). Together, these findings suggest that rILN function, spanning from minimal activity (e.g., unconsciousness) to maximal activity (i.e., optimized task engagement), provides a continuum of effective behavioral responses required of a particular task. Thus, we propose that t the rILN facilitate degrees of behavioral engagement, which we define as the application of cognitive, affective, and motor faculties required to achieve a goal (Figure 3A).

An essential component to our model of rILN function is the integration of sensory, motor, cognitive, and ARAS inputs. We propose that this culmination of afferents enables the rILN to drive behavioral engagement in a manner sensitive to changing task demands. For example, heightened rILN activity correlates to successful performance on tasks that prompt transition from low to high arousal states or require prolonged attentional engagement (Kinomura et al., 1996; Schiff et al., 2013). Thus, the rILN may be tuning behavioral engagement to optimize reward acquisition.

Through their innervation by cognitive cortical regions and re-entrant basal ganglia circuits, the rILN may exert a gain control function for cognitive and action engagement commensurate with task or goal relevance (e.g., driven by salience, internal state, and reward value). In this way, the rILN may appear to participate in attentional allocation. Traditional models describe attention as a causal filter for enhancing relevant sensory information (Broadbent, 1958). However, an alternative perspective describes attention as the consequence of competition for state representation driven by inputs conveying sensory information, prior knowledge, and internal state to the basal ganglia (Krauzlis et al., 2014). Under this framework, the rILN stand to contribute to attentional processes by relaying to the striatum an ARAS-driven signal integrating salient sensory cues and cortically-based outcome judgements. The rILN-to-striatum projection, for instance, could inform basal ganglia decision-making and, consequently, attention. Through this context-dependent enhancement of attention, or rather, influence on basal-ganglia decision-making, the rILN may facilitate the optimal engagement of cognitive resources and selection of actions to achieve reward acquisition. This action selection notion is supported by findings that rILN to striatum circuit activation elicits striatal dopamine release (Cover et al., 2019) and supports action reinforcement (Cover et al., 2019; Johnson et al., 2020). Investigation of how rILN activity and manipulations of these nuclei modulate both cortical and basal ganglia output signaling stands to inform how the rILN enhance task-dependent behavioral engagement.

This conceptual framework leads to the following testable predictions:

- Interoception: rILN activity increases with enhanced goal valuation due to interoceptive factors (e.g., unlocking a door to access a food reward in the face of hunger).
- Pain: rILN activity increases with enhanced goal valuation due to pain (e.g., unlocking a door required to escape fire).
- Social cognition: rILN activity increases with enhanced goal valuation due to complex external factors (e.g., unlocking a door to avoid an argumentative individual).
- Action expression: The rILN are engaged for both goal-directed and habitual action strategies as long as the internal or external factors driving reward acquisition are of sufficient incentive salience.
- Action learning and reinforcement: As an animal learns that a particular action leads to reward, rILN activity increases lead to further engagement in that behavior.
- Attention: Measures of executive and selective attention paid to goal-relevant cues will positively scale with increasing reward value and rILN activity.
- Conscious awareness: Increasing rILN activity correlates with decreases in attention paid to goal-irrelevant cues.
- Cognitive control: rILN inhibition evokes more pronounced deficits in tasks that require greater attentional effort or cognitive load as compared to easier versions that can be successfully completed with less engagement.

Our conceptual framework suggests that global enhancement of function is achieved through the *coordinated* activation of rILN efferents. Therefore, the results of manipulations to select rILN projections may occlude functional contributions mediated through multiple efferent targets. For example, selectively activating rILN cortical or striatal projections during sensorimotor learning may individually produce negligible or modest enhancements in performance. Activating all rILN projection neurons, however, may significantly improve learning through simultaneous excitation of striatal and cortical targets.

We predict that rILN activity manipulations may manifest in a variety of ways depending on the task. For instance, rILN activity may closely correlate with performance measures such as reaction time or accuracy, indicating fine-tuned sensitivity to behavioral outcome. Determining how the rILN activates for a particular task, in a rILN output pathway -specific manner, is poised to provide clarity for the extant data that indicates rILN signaling correlates with a range of behaviors, from saccades to reversal learning. Testing the predictions proposed here stands to elucidate the extent and limits of rILN involvement in behaviors spanning functional modality, skill-level, and attentional demand.

## Conclusion

Inspection of rILN anatomical connectivity and behavioral contributions reveals the distinct involvement of the rILN in an extensive number of functional systems. We herein propose that the rILN support a gain modulation function for adjustable engagement in goal-relevant tasks. Dysfunction in this system then would, unsurprisingly, implicate rILN pathology in a range of disorders. Future study of this system presents challenges, however. Neuroimaging resolution constraints limit investigation of the rILN in humans. *In vivo* recordings or manipulations in animals generally favor targeting of the more accessible CL nucleus. However, the robust behavioral findings from the limited interrogations of the rILN should encourage future investigation, which would benefit from rILN output-specific functional interrogation particularly centering on the understudied CM.

The high degree of integration that the rILN exhibit with many neural systems positions this area to be relevant to affective, cognitive, and action-related neuropathologies. Supporting the functional hypothesis for rILN in behavioral engagement described herein, pathological rILN activation would facilitate an overly-engaged behavioral state with a particular reward or goal, such as is the case with methamphetamine craving (Li et al., 2018). Conversely, pathological suppression of rILN activity would be predicted to give rise to cognitive and behavioral states mirroring the negative symptoms of schizophrenia (e.g., poor cognitive performance and social disengagement). Specifically targeting the rILN for therapeutic benefit may, therefore, present a range of novel therapeutic opportunities.

## Author Contributions

All authors listed have made a substantial, direct and intellectual contribution to the work, and approved it for publication.

## Conflict of Interest

The authors declare that the research was conducted in the absence of any commercial or financial relationships that could be construed as a potential conflict of interest.
